# Hepatitis B virus X protein interacts with β5 subunit of heterotrimeric guanine nucleotide binding protein

**DOI:** 10.1186/1743-422X-2-76

**Published:** 2005-08-31

**Authors:** Siew Hui Lwa, Wei Ning Chen

**Affiliations:** 1 School of Chemical and Biomedical Engineering and School of Biological Sciences, College of Engineering, Nanyang Technological University, Blk 1 Innovation Centre, 16 Nanyang Drive Unit 100 Level 1, Singapore 637722

## Abstract

**Background:**

To isolate cellular proteins interacting with hepatitis B virus X protein (HBX), from HepG2 cells infected with hepatitis B virus (HBV).

**Results:**

HBV particles were produced in culture medium of HepG2 cells transfected with the mammalian expression vector containing the linear HBV genome, as assessed by commercially available ELISA assay. A cDNA library was made from these cells exposed to HBV. From yeast two hybrid screening with HBX as bait, human guanine nucleotide binding protein β subunit 5L (GNβ5) was isolated from the cDNA library constructed in this study as a new HBX-interacting protein. The HBX-GNβ5 interaction was further supported by mammalian two hybrid assay.

**Conclusion:**

The use of a cDNA library constructed from HBV-transfected HepG2 cells has resulted in the isolation of new cellular proteins interacting with HBX.

## Background

Infection by hepatitis B virus (HBV), an enveloped DNA virus of the hepadnaviridae family, has been closely related to development of hepatocellular carcinoma (HCC). The role of this virus in the series of events leading to the onset of HCC has remained elusive [1]. However, it has been suggested that the smallest protein encoded by the HBV genome, HBX, is involved in the development of HCC [2].

Several proteins have been demonstrated to interact with the HBX protein through the use of the Yeast Two Hybrid system. These include the C-terminal portion of a novel human proteasome alpha subunit which possesses a protein sequence of close relationship to that of the 28 kD subunits from other organisms; PSMA7, the α-subunit of the 20S proteasome complex; PSMC1, the subunit of the 19S proteasome regulatory cap complex; XAPC7, a highly conserved proteasome subunit belonging to the α-subunit of the 20S proteasome complex. PSMA7, PSMC1 and XAPC7 were demonstrated to interact with the second Kunitz-type domain of the HBX protein [3-6]. Another two proteins, XAP2 (X-associated protein 2) and XIP (HBX-interacting protein) were found to be negative regulators of HBX. Overexpression of XAP2 abolished the transactivating function of HBX while the specific interaction of XIP to the carboxy terminus of HBX in differentiated HCC cells led to a reduction of wild-type HBV viral replication to levels similar to that observed after transfection with HBX-minus virus [7,8].

HBX was also shown to interact with XAP-1 (X-associated protein 1), a human homolog of the monkey UV-damaged DNA-binding protein (UV-DDB) which might be involved in DNA repair [9]. Besides these, HBX was able to interact and colocalise with HVDAC3 to the mitochondria, resulting in decreased mitochondrial transmembrane potential. HVDAC3 was identified as a new member of the human voltage-dependent anion channel (VDAC) family that provides pathways for ATP and metabolites across the mitochondrial membrane. It constitutes part of the permeability transition pore complex in the mitochondral membrane which regulates mitochondrial transmembrane potential and cytochrome c release [1].

However, these reported HBX interacting proteins have all been isolated from normal liver cDNA library (cells that had not been exposed to HBV), which may reflect physical but physiologically not meaningful interactions. There is therefore a need to comprehensively isolate and characterize, in a HBV-infected environment, cellular proteins interacting with the widely studied HBX.

Such an environment has previously been generated in vitro, firstly by showing that clonal cells derived from HepG2 transfected with a HBV-containing plasmid could elicit acute hepatitis in chimpanzees through secretion of hepatitis B surface antigen (HBsAg) particles and virions [10], and later by our investigation indicating the production of HBV particles in culture medium of HepG2 cells transfected with a replication competent HBV genome cloned in a mammalian expression vector [11].

The aim of this study is to isolate cellular proteins interacting with HBX in an HBV-infected environment.

## Results and Discussion

### Infection of HepG2 Cells by Replicative HBV Genome: an ELISA Analysis

The culture medium of the HepG2 cells transfected with infective HBV genome [11] was assayed for the presence of HBsAg prior to cell harvesting for RNA extraction. In Table 1, values D1, E1, F1, G1, H1, A2, B2, C2, D2, E2, F2, G2 and H2 corresponded respectively to the undiluted medium, its 10×, 10^2^×, 10^3^×, 10^4^×, 10^5^×, 10^6^×, 10^7^×, 10^8^×, 10^9^×, 10^10^×, 10^11^×, 10^12^× serial dilutions measured at an absorbance of 450 nm. The presence of HBsAg was indicated in the undiluted medium, its 10 × dilution and up to 10^4 ^× dilution. The values of these samples were respectively, 2.393, 0.464 and down to 0.186. They exceeded the calculated cut-off of 0.179, thus indicating the presence of HBsAg. The results of this assay run were valid as the criteria for the quality control had been met. The mean A_450 _of the negative controls was 0.129, which did not exceed 0.2. The A_450 _of the positive control was 1.494, which was 1.365 higher than the mean A_450 _of the negative controls. The value of the positive control fulfilled the quality control criteria which requires the value to be at least 0.8 higher. The cut-off of the assay reading, 0.179, was derived by adding 0.05 to the mean A_450 _of the negative controls. Based on this cut-off value, readings that were equivalent to or higher than 0.179 indicated reactive samples. Taken together, our results indicated the presence of viral infection process in HepG2 cells transfected with the replicative HBV genome.

**Table 1:** Production of HBV in Transfected HepG2 Cells by ELISA Analysis

Wells	Samples	Absorbance at 450 nm (A_450_)
A1	Negative Control 1	0.131
A2	Negative Control 2	0.126
A3	Positive Control	1.494
A4	Undiluted growth medium of HBV-transfected HepG2 cells	2.393
A5	10 × diluted growth medium	0.464
A6	10^2 ^× diluted growth medium	0.164
A7	10^3 ^× diluted growth medium	0.142
A8	10^4 ^× diluted growth medium	0.186

### cDNA Library Construction

**Figure 1:**
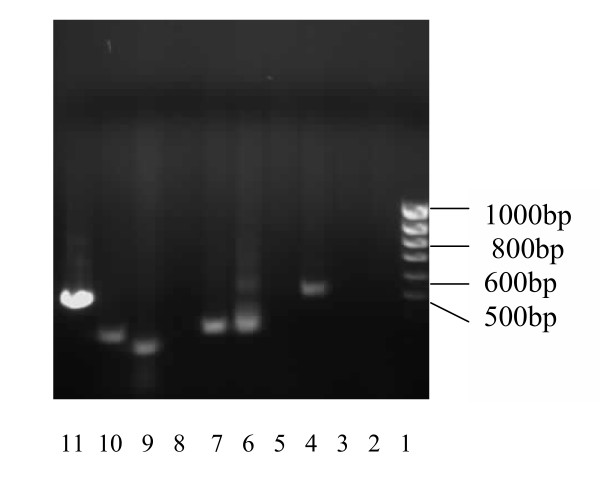
**Analysis of Insert Size of the cDNA Library from HBV-transfected HepG2 Cells**. Lane 1: 100 bp Marker. Lanes 2–11: Yeast plasmid DNA extracted from library colonies to check for range of sizes of inserts before use of library for screening. Lanes 4, 6, 7, 9, 10 and 11 showed inserts ranging between 300 to 600 bp.

The cDNA library that would be used in the yeast-two-hybrid screening for protein-protein interactions was constructed by simultaneous transformation of double-stranded cDNA and the activation domain vector, pGADT7-Rec, into yeast strain AH109. After a 4-day incubation period, yeast plasmid DNA was extracted 10 random yeast colonies used as template for PCR. Presence of cDNA inserts of varied sizes ranging between 300 to 600 base pairs were indicated in lanes 4, 6, 7, 9, 10 and 11 as shown in Figure 1. Library transformants were then harvested and pooled.

### Yeast Two Hybrid Screening for HBX Interacting Proteins

The total number of colonies that resulted from the yeast two hybrid screening and the percentage number of colonies that progressively developed a blue colour over a span of 4 days was tabulated in Table 2. The absence of blue color for the 10 colonies resulting from the empty pGBKT7 suggested that 50% of the blue colonies corresponded to HBX-interacting proteins.

**Table 2:** Summary of Yeast Two Hybrid Screening for HBX Interacting Proteins

Transformed Y187 yeast strain	Total number of colonies after interaction with cDNA library	% number of blue colonies			
		
		Day			
		
		1	2	3	4
Full length HBX in pGBKT7	1.6 × 10^4^	0	10	20	50
pGBKT7 without HBX	10	0			

Blue colour development was due to the use of the X-α-Gal assay system as an indication of positive interactions in the yeast-two-hybrid system. Colonies that resulted from the screening of the cDNA library and the HBX-pGBKT7 bait as well as those that resulted from the negative control reaction which is the interaction between the empty bait and the cDNA library were observed for their development of blue colouration on SD/-Ade/-His/-Trp/-Leu/X-α-Gal agar.

**Figure 2:**
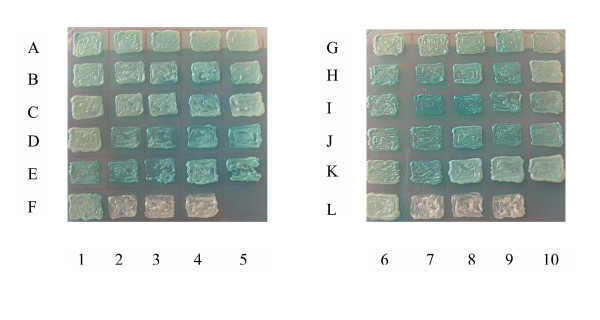
**Positive Yeast Colonies with Potential HBX Interacting Proteins**. Comparison between control yeast colonies and positive colonies from the library screening that developed blue colour over a 5-day incubation period at 30°C. A1-5, B1-5, C1-5, D1-5, E1-5, F1, G6-10, H6-10, I6-10, J6-10, K6-10, L6: colonies that turn blue after α-galactosidase assay screening indicated positive interaction between bait and cDNA library. F2 and L7: control using bait plasmid cloned with HBX transformed into yeast strain Y187 to test if the bait alone led to transcriptional activation. α-galactosidase assay screening indicate negative interaction between bait and library. Colonies remained white. F3 and L8: control using negative control plasmid, pGBKT7-Lamin transformed into yeast strain Y187. α-galactosidase assay screening indicate negative interaction between bait and library. Colonies remained white. F4 and L9: Control using bait plasmid, pGBKT7 transformed into yeast strain Y187. α-galactosidase assay screening indicate negative interaction between bait and library. Colonies remained white.

Y187 yeast strain was transformed individually with each of the 3 control bait constructs empty pGBKT7 vector, full length HBX in pGBKT7 vector and negative pGBKT7-Lamin control vector. Colonies that grew on SD/-Trp were transferred to SD/-Trp/X-α-Gal. Blue colour development was observed over a period of 5 days. Transformation of these 3 negative control plasmids into Y187 yeast strain did not produce yeast colonies on SD/-Ade/-Trp and SD/-His/-Trp plates after incubation of 5 days. A comparison of the intensity of blue coloration of positive clones resulting from the library screening is shown in Fig 2. In contrast to these blue colonies, only white color was observed for the three negative control clones from the individual transformation of the empty pGBKT7 vector, full length HBX in pGBKT7 vector and negative pGBKT7-Lamin control vector.

The negative control experiment as shown in Table 2, in which an empty bait vector was used in place of the HBX bait yielded 10 colonies after the screening but none of these turned blue. Therefore, it can be concluded that the X-α-Galactosidase assay is a reliable method for determining positive interactions in the yeast-two-hybrid system. As shown in Fig. 2, the HBX bait alone did not activate transcription of the reporter gene that led to blue colour development. This confirms that the blue colonies which resulted after screening were indeed due to interactions between the AD fusion protein and the BD fusion protein in the two separate yeast strains.

Through the analysis of sequences of 9 positive clones, only one had a library insert that was in frame with the activation domain of GAL4 protein on pGADT7-Rec vector. The insert corresponded to human guanine nucleotide binding protein β subunit 5L (GNβ5). Although it has been well established that HBX interacts with many molecules of cellular signaling pathway, GNβ5 has hitherto not been identified as a HBX-interacting protein. The use of a cDNA library constructed from HBV-transfected HepG2 cells would therefore be significant in that it would enable cellular signaling events occurring after HBV infection to be traced via interactions with the HBX protein which had been implicated in HBV-related hepatocellular carcinoma. This also implies that the use of an infected library increases the likelihood of identifying new interacting partners.

Despite the stringency of nutritional selection through the use of the AH109 yeast strain which contains three reporters, namely, *ADE2*, *HIS3 *and *MEL1*, the yeast-two-hybrid system is only able to reduce the number of false positives. There still exists an uncertainty of whether interactions are always true positives. For this reason, the Mammalian-Two-Hybrid system was used to re-confirm the interaction. This system verifies protein-protein interactions by transcriptional activation. Similar to the yeast two hybrid system, the Mammalian-Two-Hybrid Checkmate system consists of two vectors namely pBIND and pACT. Interaction between two proteins expressed from these two vectors will be assessed by the luciferase activity following the transient transfection of both vectors into mammalian cells.

**Figure 3:**
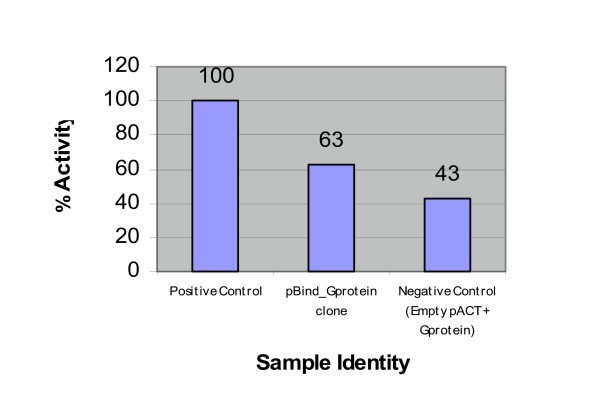
**Dual-Luciferase Reporter Assay demonstrating the interaction between GNβ5 and HBX**. The pBIND-GNβ5 plasmid was transfected together with the pACT-HBX plasmid and the pG5luc luciferase vector to test the protein-protein interaction between partial GNβ5 sequence and full-length HBX in the Mammalian-Two-Hybrid system. The percentage activity as reflected by the luciferase reporter was plotted out in the graph.

The pBIND-GNβ5 plasmid was transfected together with the pACT-HBX plasmid and the pG5luc luciferase vector to test the protein-protein interaction between partial GNβ5 sequence and full-length HBX in the Mammalian-Two-Hybrid system. The percentage activity as reflected by the luciferase reporter was plotted out in the graph shown in Figure 3. The assay value given by this interaction was compared against the positive control nteraction between pACT-MyoD and pBIND-1d and a negative control which included pBIND-GNβ5 and the empty pACT vector. The results showed that the luciferase activity triggered by interaction between the partial GNβ5 and full length HBX was twenty percent higher than that produced by the negative control reaction, thus suggesting presence of interaction. However, the luciferase activity of the interaction was only 63% of that measured for the interaction in the positive control. Thus, although the experimental value exceeds that of the negative control, the confirmation of the interaction between HBX and partial GNβ5 can be fine tuned by further tests using the same system.

### Possible Role of GNβ5 in HBX-mediated Cellular Signaling Pathway

G proteins are heterotrimeric guanine nucleotide binding proteins that are involved in signal transduction. They are peripherally associated with the plasma membrane and function to couple signals to seven transmembrane-spanning surface receptors. G proteins consist of α, β and γ subunits, of which β and γ subunits are tightly associated. In a typical G protein coupled signaling pathway, the ligand-activated receptor catalyzes the exchange of guanine nucleotides in the α subunit. The GTP-bound α subunit dissociates from the receptor as well as the βγ subunit and proceeds to activate its respective effector molecule. The free βγ subunit also activates a similar or different effector molecules.

The hydrolysis of the bound-GTP to GDP by intrinsic GTPase activity of the α subunit leads to a conformational switch, resulting in the termination of its effector interaction. The resulting α-GDP re-associates with the free βγ subunit. This is followed by re-entry of the newly formed heterotrimer into the signaling cycle. To date, 17 α subunits, five β subunits and 12 γ subunits have been identified [12].

The GNβ5 isolated in our study would be of significance. Previous reports had suggested that the GNβ5 protein was essentially soluble. The GNβ5 protein displays sequence homology to a group of Gβ-like proteins known as the WD-40 repeat family [13]. It has been proposed that this repeat enables G protein β subunits to adopt multiple conformations which could greatly expand the number of signaling partners.

### G proteins and Cancer Development

G proteins have also been implicated in cancer development [12]. In the classical paradigm, GPCR-mediated signal transduction involves the agonist-dependent interaction of GPCRs with G proteins at the plasma membrane, and the subsequent generation of soluble second messengers or ion currents by membrane localized effectors.

There exist two major mechanisms for transmembrane signaling in intercellular communication, mediated respectively by receptor tyrosine kinases and by GPCRs. Recent evidence shows that the two pathways can converge on the same effectors, for example, Ras and mitogen-activated protein kinase (MAPK). Both systems appear to use specific protein-protein interactions for localization of key signaling intermediates to appropriate membrane compartments. For receptor tyrosine kinases, protein-protein interactions are mediated by Src homology SH2 and SH3 while for GPCRs, interaction of the βγ complex of heterotrimeric G proteins [13].

Based on the above, HBX probably associates with GNβ5 at the early cellular stage of HBV infection. From previous reports that Ras and MAPK constitute the point of convergence of the tyrosine kinase and the G protein coupled receptor signaling pathways, it is likely that HBX plays a role in bridging and activating the Src-kinase and MAPK mediated pathways at the early stage of viral infection. Further functional studies, including the down-regulation of expression of GNβ5 using siRNA, should shed new lights on its role in HBV infection.

## Methods

### Cell Culture and Transfection

HepG2 cells were grown in Dulbecco's Modified Eagle's Medium (DMEM) (Gibco) supplemented with 10% fetal calf serum. HepG2 cells cultured in 10 ml of DMEM in a 9 cm × 6 cm flask were transiently transfected by Effectene (QIAGEN) with 1.5 μg of pcDNA 3.1-HBV DNA when cell confluence reached 60%. Cells were maintained in a saturated, humidified environment of 5% CO_2 _– 95% air at 37°C. After 36 hours of incubation, 1 ml of the cell culture medium was retained for ELISA analysis.

### ELISA Analysis

The HBV DNA level in the culture medium of the transfected HepG2 cells was analysed by ELISA (Murex HBsAg Version 3, ABBOTT).

### Bait Plasmid Construct

The full length HBX gene sequence of 462 base pairs was amplified by PCR using full length HBV DNA (*adw2 *subtype) as template. Oligonucleotide primers were designed with NcoI and PstI enzyme restriction sites as follows:

5'-TGCCATGGCAATGGCTGCTAGGCTGTACTGCC-3'

5'-AACTGCAGTTAGGCAGAGGTGAAAAAGTT-3'

The PCR product and the binding domain vector, pGBKT7 of 7.3 kb (MATCHMAKER Two-Hybrid System 3, BD Biosciences) were digested individually with enzymes, NcoI and PstI. (New England Biolabs) After each enzymatic digestion, the PCR product and vector were column-purified. (QIAGEN) 2 μl of the purified PCR product was ligated to 6 μl of the binding domain vector in a reaction mixture which included 1 μl of T4 DNA Ligase and 1 μl of T4 DNA Ligase Buffer (New England Biolabs). Incubation of the mixture was carried out overnight at 16°C. 5 μl of the ligation mixture was transformed into 50 μl of DH5α competent cells (Stratagene). Transformed cells were plated on 30 μg/ml Kanamycin LB agar plates and incubated at 37°C. Colonies were analysed for presence of correctly inserted bait DNA using the pair of primers used for amplifying the corresponding HBX bait DNA in a PCR reaction. Plasmid DNA from the colony identified to contain the correct insert was extracted using the Miniprep Plasmid DNA extraction kit (QIAGEN) and was transformed into yeast strain, Y187 (Clontech Laboratories, Inc.)

### cDNA Library Construction

RNA was isolated from 2 × 10^7 ^HBV-transfected HepG2 cells in a final elution volume of 50 μl using the RNeasy Mini Kit (QIAGEN). cDNA was synthesised using the MATCHMAKER Library Construction and Screening Kit (Clontech Laboratories, Inc) First strand cDNA synthesis was carried out using 1 μl of a random CDS III/6 Primer: 5'-ATTCTAGAGGCCGAGGCGGCCGACATG-NNNNNN-3', 2 μl of RNA and 1 μl of water. The mixture was incubated at 72°C for 2 minutes followed by incubation on ice for 2 minutes. This was followed by the addition of 2 μl of 5 × First-Strand Buffer, 1 μl of DTT (20 mM), 1 μl of dNTP mix (10 mM) and 1 μl of Moloney Murine Leukemia Virus Reverse Transcriptase. This was followed by a 10 minute-incubation period at 25°C and a 10 minute-incubation period at 42°C. 1 μl of SMART III Oligonucleotide (10 μM; 5'AAGCAGTGGTATCAACGCAGAGTGGCCATTATGGCCGGG-3') was addedand the reaction mixtures were incubated at 42°C for 1 hour. The tube was incubated at 75°C for10 minutes to terminate first-strand synthesis and cooled to room temperature before adding 1 μl (2 units) of RNaseH. Next, incubation at 37°C was carried out for 20 minutes. Second strand cDNA was synthesised by Long Distance-PCR. Sufficient double-stranded cDNA was prepared for transformation by setting up two 100 μl reaction mixtures, each composed of the following: 2 μl of first-strand cDNA, 70 μl of deionised water, 10 μl of 10 × Advantage 2 PCR Buffer, 2 μl of 50 × dNTP mix, 2 μl of 5' PCR Buffer, 2 μl of 3' PCR Buffer, 10 μl of GC-Melt Solution and 2 μl of 50 × Advantage 2 Polymerase Mix. Thermal cycling was carried out using the following conditions: 95°C for 30 seconds; 24 cycles of 95°C for 10 seconds, each followed by 68°C for {6 minutes + [5(x)] seconds} where x increased per cycle from "0, 1, 2, 3,..." to 23; 68°C for 5 minutes. Double-stranded cDNA was column-purified using CHROMA SPIN + TE - 400 columns (Clontech Laboratories, Inc.).

200 μl of AH 109 yeast competent cells (BD Biosciences) was transformed with 14 μl of ds cDNA and 6 μl of pGADT7-Rec. The transformation mixture was distributed onto 130 100 mm SD/-Leu agar plates. The plates were incubated at 30°C for 5 days.

### Harvesting of Yeast Transformants

1 litre of freezing medium (YPD medium with 25% v/v glycerol and sterilised at 121°C for 15 minutes) was prepared for the harvest of transformants. 5 ml of freezing medium was added to each plate and yeast colonies were scraped off the plates and pooled into a sterile 1 litre conical flask. The mixture was mixed well by swirling the flask before storing as 1 ml aliquots at -80°C.

### Interaction of Yeast Strains

A single yeast colony from the transformation of the bait plasmid into Y187 yeast strain was innoculated into 50 ml of SD/-Trp liquid medium and incubated at 30°C with shaking at 270 rpm. When OD_600 _of the culture reached 0.8, it was combined with an 1 ml aliquot of the cDNA library in a 1-litre conical flask together with 45 ml of yeast culture medium (2 × YPDA medium with Kanamycin, 50 μg/ml). The mixture was incubated for 24 hours at 30°C with gentle swirling at 40 rpm.

A drop of the mixture was analysed under phase-contrast microscope (400×) to check for the presence of zygotes. Centrifugation of the mixture was carried out at 1000 × g for 10 minutes and the supernatant was discarded. The cell pellet was resuspended in 10 ml of 0.5 × YPDA/Kanamycin (50 μg/ml). The entire mixture was plated out onto 100 mm SD/-Ade/-His/-Leu/-Trp agar plates. 100 μl of the suspension was distributed evenly onto each plate. The plates were incubated at 30°C for 5 days.

### Selection of Yeast Diploids Expressing Interacting Proteins

Only colonies that measured 2 mm or more after 5 days of incubation at 30°C were selected for further screening. 60 colonies were randomly selected for the first round of screening. These were streaked onto SD/-Leu/X-α-Gal agar plates. The agar plates were incubated at 30°C over a 4-day incubation period. 52 colonies that turned blue at the end of the 4-day incubation period on the SD/-Leu/X-α-Gal agar plates were individually innoculated into 10 ml of SD/-Leu medium and 5 ml of SD/-Ade/-His/-Leu/-Trp medium. The liquid cultures were incubated with shaking at 30°C, 280 rpm. After 48 hours, glycerol stocks of these cultures were prepared by adding 1.4 ml of each culture to 0.3 ml of glycerol and 0.3 ml of the respective medium. The stocks were stored at -80°C. The SD/-Leu cultures were centrifuged at 5500 rpm, 4°C, for 10 minutes. Yeast plasmid extraction was carried out to determine library insert sizes. 2 μl of extracted plasmid was used as template for PCR. 52 samples corresponding to bands with sizes of approximately 500 basepairs or more were purified using PCR purification columns (QIAGEN) and sequenced. These 52 colonies were also streaked onto SD/-Trp/X-α-Gal agar plates in a grid-like pattern. The following plasmids, the pGBKT7 vector, pGBKT7 cloned with full-length HBX and the pGBKT7-Lamin negative control vector (BD Biosciences) were transformed into Y187. The resulting colonies were used as controls. The agar plates were incubated at 30°C and the rate at which the colonies turned blue over a 4-day incubation period was noted.

### Confirmation of Interaction by Mammalian CheckMate System

The partial human guanine nucleotide binding protein β subunit 5L (GNβ5) insert was amplified for cloning in-frame with the GAL 4 containing binding domain vector, pBIND (CheckMate System, Promega) for use in the Mammalian-two-hybrid system. The 5' and 3' primers were designed with BamHI and EcoRV digestion sites respectively as follows: 5'-GAGGATCCTCAAAGATAAGAGGAGGATCGT-3' and 5'-GAGATATCTCGGGGGCCAGGTCCAAGCAGA-3'

HBV DNA (*adw2 *subtype) was used as template for PCR to amplify the full length HBX sequence of 462 base pairs using the following 5' and 3' primers which were designed with SalI and EcoRV digestion sites respectively:

5'-TGGTCGACCAATGGCTGCTAGGCTGTACTGC-3'

5'-AAGATATCTTTTAGGCAGAGGTGAAAAAGTT-3'

The resulting PCR product was purified and cloned into the Herpes Simplex Virus VP16 activation domain vector, pACT, of the Mammalian-two-hybrid system (CheckMate System, Promega).

### Transfection of HepG2 Cells

Transfection of HepG2 cells was carried out using Effectene reagent (QIAGEN) when cells reached 35% confluence. Duplicated reactions were carried out in two 6-well plates. For each of the positive control, negative control and experimental wells, 1.2 ng of combined DNA composing of 3 different plasmids in a 1:1:1 ratio was used. The positive control reaction consisted of 0.4 ng of each of the following vectors, pG5*luc*, pBIND-1d and pACT-MyoD. The negative control reaction consisted of 0.4 ng of each of the following vectors, pG5*luc*, empty pACT vector and the GNβ5-pBIND vector. The actual experimental reaction to confirm the interaction consisted of 0.4 ng of each of the following vectors, pG5*luc *GNβ5-pBIND vector and the HBX-pACT vector. Cells were incubated in a saturated, humidified environment of 5% CO_2 _– 95% air at 37°C. After 2 hours of incubation, the growth medium was aspirated and the cells were washed twice using 2 ml of PBS per wash. 2 ml of fresh medium was added to each well and the plates were incubated in 5% CO_2 _and 95% air at 37°C for 36 hours.

### Harvesting and Lysis of Transfected cells

The transfected cells were trypsinised and the cell suspension of each well was collected and centrifuged at 1500 rpm at 4°C for 3 minutes. The supernatant was removed and each cell pellet was resuspended in 4 ml of PBS. Centrifugation was then carried out at 1500 rpm at 4°C for 3 minutes. The supernatant was discarded and 250 μl of 1 × Passive Lysis Buffer (Promega) was used to lyse each cell pellet.

### Dual-Luciferase Reporter Assay

20 μl of each test sample was mixed with 100 μl of Luciferase Assay Reagent II (Promega) in a luminometer tube. The luminometer (Sirius Tube Luminometer, Berthold Detection Systems) was programmed to provide a 2-second pre-read delay, followed by a 10-second measurement period for each reporter assay. Upon recording the first reading, 100 μl of 1 × Stop & Glo Reagent (Promega) was promptly added to the reaction mix. After mixing, the second reading was recorded.

## Competing interests

The author(s) declare that they have no competing interests.

## Authors' contributions

SH Lwa was a recipient of a graduate research scholarship from Nanyang Technological University, and conducted experiments under the direction of Dr. Chen. Dr. Chen initiated the research, writing of the draft manuscript with subsequent editing and revisions by both authors.
